# Soluble microbial products (SMPs) release in activated sludge systems: a review

**DOI:** 10.1186/1735-2746-9-30

**Published:** 2012-12-18

**Authors:** Hamed Azami, Mohammad Hossein Sarrafzadeh, Mohammad Reza Mehrnia

**Affiliations:** 1 Biotechnology Group, School of Chemical Engineering, College of Engineering, University of Tehran, Tehran, Iran

**Keywords:** Soluble microbial products (SMPs), Effluent, Biodegradability, Wastewater, Activated, Sludge

## Abstract

This review discusses the characterization, production and implications of soluble microbial products (SMPs) in biological wastewater treatment. The precise definition of SMPs is open to talk about, but is currently regarded as “the pool of organic compounds that are released into solution from substrate metabolism and biomass decay”'. Some of the SMPs have been identified as humic acids, polysaccharides, proteins, amino acids, antibiotics, extracellular enzymes and structural components of cells and products of energy metabolism. They adversely affect the kinetic activity, flocculating and settling properties of sludge. This review outlines some important findings with regard to biodegradability and treatability of SMPs and also the effect of process parameters on their production. As SMPs are produced during biological treatment process, their trace amounts normally remain in the effluent that defines the highest COD removal efficiency. Their presence in effluent represents a high potential risk of toxic by-product formation during chlorine disinfection. Studies have indicated that among all wastewater post-treatment processes, the adsorption by granular activated carbon combined with biologically induced degradation is the most effective method for removal of SMPs. However, it may be concludes that the knowledge regarding SMPs is still under progress and more work is required to fully understand their contribution to the treatment process.

## Introduction

Effluents from biological wastewater treatment systems contain a variety of colloidal and soluble organic compounds, including residual degradable or hard-biodegradable influent substrate, intermediates and end products, complex organic compounds formed through chain reactions with both intermediate and final degradation products categorizing as soluble microbial products (SMPs). The presence of complex residual microbial products in wastewater effluents was confirmed from the time when Gaffney and Heukelekian conducted a study dealing with comparison of oxidation rates of the lower fatty acids under various conditions [1]. Since then many researchers [2-5] have shown that the majority of the soluble organic materials in effluents produced through biological treatment processes are actually microbial products (SMPs). Their presence is an issue of great interest not only in terms of achieving current discharge standards, but also because they effectively set the lower boundary for treatment.

**Figure 1:**
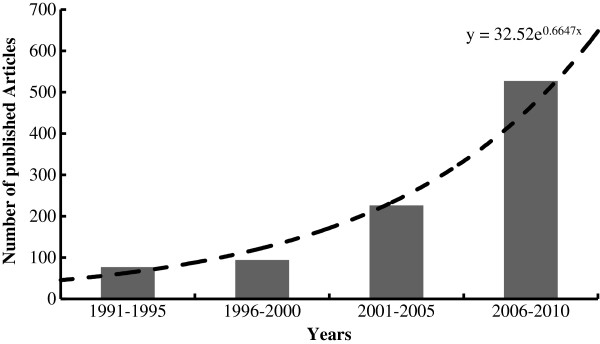
Number of published articles on SMP and related subjects in various years extracted from Engineering-Village databases.

Since the application of membrane based technologies in wastewater treatment and its combination with biological processes in a system such as membrane bioreactor (MBR), more special attention was given to the SMPs due to their role in membrane fouling. Nowadays, many researches concentrate on SMP and their effect on performance of biological processes as the increasing amount of published articles during last two decades confirm it (Figure 1).

Paying attention to the presence of SMPs, has resulted in development of wastewater treatment systems. Previously, models of wastewater treatment systems had been based on the Monod models which predict the effluent composition of the rate limiting substrate independently of the influent substrate concentration. Monod models did not agree with experimental results and the interaction of SMPs formation paved the way for more accurate modeling of wastewater treatment [6].

Activated sludge models (ASMs) proposed by IWA are fundamentally based on first engineering principles, meaning that the model equations were developed from general balance equations applied to mass and other conserved quantities, resulting in a set of differential equations.

Many researchers tried to improve the activated sludge models by adding SMPs components into ASM1 and ASM3, and also by integrating the extracellular polymeric substances (EPSs) into ASM1 model [7-10].

Yoon *et al*. [11] proposed a kinetic model to calculate the sludge production and aeration requirement. Tian *et al*. [4] extended ASM3 model to ASM3-SMP with taking into account the concept of simultaneous storage and growth of SMPs by considering two components: Utilization associated product (S_UAP_) and Biomass associated products (S_BAP_). However, all of them (except [4,7]) used parameters directly taken from literature derived from activated sludge systems or even biofilm systems.

These advances in modeling, resulted in a better understanding of how mixed bacterial populations work. Chemical structure of SMP compounds has also been clarified due to advances in chemical identification and analytical methods. Most of the works have focused on SMPs in aerobic systems, but some of researches have also investigated SMPs in anaerobic systems [12-14].

As mentioned above, SMPs are the main portion of contaminants in effluent of activated sludge not only increasing the BOD content of effluent, but also deteriorating settling properties of activated sludge. In addition, researches indicated that SMPs act as glue and bind the suspended flocs causing increase in activated sludge viscosity [15,16]. Furthermore, SMPs as a kind of biopolymers have detrimental impact on activated sludge process [17]. The main obstacle to evaluate the impact of SMPs in wastewater treatment process is the difficulty in SMPs measuring especially in complex effluents [18].

Many works have been conducted on pure cultures and have defined feeds for better understanding and evaluation the effects of SMPs on treatment process. However, in industrial systems the phenomenon is more complicated and can be considered as an uncompleted task that needs to be studied more.

Despite the obvious importance of SMPs in wastewater treatment processes, a few publications have attempted to summarize all the information in a comprehensive review. Kimura *et al*. [19], performed a precise study of microbial product formation in biological systems which concentrated on: the measured characteristics of SMPs in biological processes; effect of sludge age; and the literatures on product formation and effect of product on membrane fouling in MBR. The objective of present paper is to review the currently available literature on SMPs, focusing on: their definition and their origin; biodegradability properties; the factors affecting the production of SMPs; and the treatment (i.e. removal) of SMPs. The current state of the art will be summarized and, finally, a closing section will address the future research needs with regard to SMPs.

### What are SMPs?

It is well known that microorganisms produce organic substances during substrate degradation, growth and endogenous decay. The term SMPs as biological produced organic material, still, has been used by many authors without precise definition. This is rather due to the difficulty in identifying SMPs formation procedures and also to the complexities of effluent composition and especially tracing the origin of the mixture of compounds in a biological treatment system [20]. Boero *et al*. [21] stated that SMPs result “from intermediates or end products of substrate degradation and endogenous cell decomposition”, while most of the studies define SMPs as “the pool of organic compounds that result from substrate metabolism and biomass decay during the complete mineralization of simple substrates” [3,13,22-24]. The inclusion of “during the complete mineralization of simple substrates” in the definition is open to some debate.

SMPs are sometimes the hard-biodegradable end-products of the incomplete microbial degradation of more complex compounds that can act as the substrates for another group of microorganisms in the aerobic or anaerobic chains. Chudoba [25] classified the SMPs produced by activated sludge microorganisms into three categories:

1. Compounds excreted by microorganisms owing to their interaction with the environment.

2. Compounds produced as a result of substrate metabolism and bacterial growth.

3. Compounds released during the lysis and degradation of microorganisms.

This classification system is used in wastewater process design, particularly from a process engineering point of view, but it does not address the biodegradability of non intermediate soluble microbial products. Engineers prefer Chudoba’s classification whereas microbiologists tend to classify SMPs formation into three categories: growth-synonymous; growth-associated and growth-independent [26,27]. If we apply this classification to organic compounds produced by activated sludge, then growth-independent products are located in categories 1 and 3 of Chudoba’s classification while growth-synonymous and growth-associated products are placed in second category of Chudoba’s classification.

However recent researches classified the SMPs into two different categories based on the bacterial phase from which they were derived [28,29]:

1. Utilization associated product (UAP), i.e. SMPs that are associated with substrate metabolism and biomass growth and are produced at a rate proportional to the rate of substrate utilization.

2. Biomass associated products (BAP), i.e. SMPs that are associated with biomass decay and are produced at a rate proportional to the concentration of biomass.

Using these categories they were able to successfully model substrate utilization, SMP formation and the removal of total soluble organic matter in biological treatment processes [4,30].

### The origin of SMPs

The complete list of the origin of SMPs is provided by Kuo [31]. He mentions the following factors as causes of SMPs release:

1. Concentration equilibrium: organisms excrete soluble organic materials to maintain concentration equilibrium across the cell membrane [32,33]. Concentration equilibrium state chemical potential equilibrium which is the main cause of conditional stress around the cells that cause to excretion of soluble organic materials [34,35]. Nossal et al. [36] indicated that microorganisms secrete SMPs in highconcentrations of salt to protect against osmotic pressure.

2. Starvation: bacteria SMPs during starvation as they must obtain energy for maintenance by metabolism of intracellular components or endogenous respiration when the substrate is essentially absent [37,38]. Scientists considered all content of SMPs producing in starvation condition as biomass-associated products (BAP) and tried to investigate the impact of them on biological treatment system performance [4,39].

3. Presence of energy source: the presence of an increased concentration of exogenous energy source can stimulate the excretion of SMPs [40].

4. Substrate-accelerated death: sudden discharge of a carbon and energy source to bacteria starved for carbon and energy may accelerate the death of some bacteria which results in production of SMPs [41].

5. Nutrients deficiency: if essential nutrients are present in very low concentrations, SMPs may be produced to scavenge the required nutrient [42].

6. Environmental stress: SMPs are produced in response to environmental stress, such as extreme temperature changes [43], pH variation [26], osmotic shocks [44],and salinity [45,46]. Other researchers also speculate that SMPs are produced in response to toxic substances such as heavy metals [31,47].

7. Normal bacterial growth and metabolism: SMPs, such as extracellular enzymes, are not only produced during stressed conditions but also during normal growth and metabolism, especially degradation of biodegradable hydrocarbons [48]. Amani *et al*. [49], furthermore, produced SMPs as a surfactant through the biological degradation of synthetic feed containing whey, crude oil and sucrose.

8. Endogenous decay: as mentioned SMPs have been classified into two groups based on the bacterial phase in which they are derived: the utilization associated products (UAP) derived during the original substrate in microbial growth and the biomass-associated products the BAPs generated in the endogenous phase [50].

### The characteristics of SMPs

Bulk parameters that state overall performance of a treatment system in regard to SMPs have been studied well. The most of researches have focused on the global characteristics of SMPs such as distribution of molecular weights (MW), biodegradability and toxicity [51]. SMPs have a wide range of MW; determination of MW distribution of SMPs sometimes could be very informative and even help evaluating the efficiency of treatment process [52]. There is no standard method for determining the MW distribution of soluble organic compounds. This creates complexities in comparing results from different studies and only their relative MW measurements and trends could be evaluated. Materials size distributions of soluble organic compounds are determined either as a continuous distribution using gel permeation chromatography [53] or as a discrete distribution using ultrafiltration UF membranes in stirred cells [54]. The sizes of these soluble organics are referred to as apparent molecular weight in comparison with standard compounds of known MW [55].

Biodegradability of SMPs is important because it can present the effluent environmental risks and BOD level. Residues of SMPs in effluent could induce the formation of several toxic materials during some post-treatment steps such as chlorine disinfection.

SMPs are also characterized with considering the proportion of protein base SMP (SMP_p_) and carbohydrate base SMP (SMP_c_). While SMP_p_ has generally a hydrophobic tendency, SMP_c_ is more hydrophilic. Total organic carbon (TOC) level and more rarely specific ultraviolet absorbance (SUVA) are among the other measurements that can help SMP characterization. Aromaticity and hydrophobicity of SMPs can be determined by the measurement of the SUVA.

Zeta (ζ) potential and hydrophobicity can state electrostatic interaction of SMPs with membrane surface in MBRs and flocculation potential in activated sludge treatment process [56].

### Treatment of SMPs

SMPs concentration in wastewater influent is negligible and they are generally produced during wastewater treatment process. This particularity makes their removal process different from other organic pollutants. Removal of SMPs from biological wastewater treatment systems is in theory possible using advanced techniques such as MBR; however it is inevitable that some SMPs remain in the effluent. SMPs are necessary for flocculation in activated sludge process [57], but they induce some inconvenient consequences in treatment process. The part of remained SMPs in effluent results in organic material discharge to the environment. Post- treatment method should be considered in treatment processes to remove the hard-biodegradable SMPs. These SMPs are also precursors for chlorinated organic compounds such as trihalomethanes (THMs) formation during effluent chlorination [27]. Several researchers have studied the treatability of SMPs with complementary treatment techniques, such as activated carbon adsorption and coagulation [58,59]. Associated literatures with these mentioned techniques for removing SMPs have been listed in Table 1.

**Table 1:** Various techniques studied for treatment of SMPs and their corresponding literature

**Treatment processes**	**Reference**
Adsorption by activated carbon	[58,60]
Filtration by membrane	[61,62]
Adsorption by synthetic resin	[63,64]
Ozonation	[65,66]
Coagulation and electro-coagulation	[45,67]
UV treatment	[68,69]

Reviewing all of these literatures indicates that adsorption on granular activated carbon (GAC) combined with biologically induced degradation was the most effective method for removal of SMPs [70]. There are several investigations trying to improve conventional activated sludge process to reduce biopolymers content in effluents from biological treatment [71,72]. Parkin and McCarty [73] applied several methods to remove soluble organic nitrogen from effluents, and found that the most efficient treatment process was granular activated carbon (GAC) adsorption (85% removal) and chemical precipitation using high concentrations of ferric chloride (70% removal). Afterward, Randtke and McCarty [74] carried out the feasibility study on diverse individual and combined wastewater treatment processes for the removal of residual soluble organics from aerobic treatment process effluent and again came to the conclusion that GAC adsorption was generally the most proficient. Schultz and Keinath [75] observed that nearly 50% of SMPs were adsorbed onto powdered activated carbon PAC, but only 4% of the adsorbed SMPs were biodegradable that demonstrates the refractory nature of the SMPs. Guo et al. [76] recently, proposed improved biological activated carbon (BAC) which is the combined O_3_-BAC and AC/O_3_-BAC processes to remove refractory organic matter from treated sewage effluent. They found that maximum dissolved organic carbon (DOC) removal efficiency was 40% during steady state ozonation step. In comparison with O_3_-BAC system, the AC/O_3_-BAC system degraded the effluent DOC more significantly.

Removal of SMPs produced in anaerobic wastewater treatment systems is more complicated. Post- treatment of anaerobic effluents with activated carbon conducted by Barker *et al*. [60], showed that low MW materials (i.e. MW<1 kDa) from anaerobic treatments were the most difficult to be adsorbed on GAC.

## Conclusion

Despite all of these mentioned studies, the knowledge regarding SMPs is still under progress and more works is required to fully understand their contribution in each biological treatment process. However, based on this review the current state of the art on SMPs can be summarized as the following main points:

● As a result of the complicated measuring procedures of SMPs, their definitions are somewhat uncertain and depend on what point of view is taken. The most widely accepted definition for SMPs which comes from an engineering perspective is “organic compounds produced during microorganism metabolism and biomass decay”. A basic operational definition is any soluble material that appears in the effluent while it was not present in the influent.

● SMPs have been classified into two groups: substrate utilization associated products and biomass associated products. UAPs are associated with substrate metabolism and biomass growth and are produced at a rate proportional to the rate of substrate utilization, while BAP are associated with biomass decay and are produced at a rate proportional to the concentration of biomass.

● SMPs are hardly biodegradable and the kinetic of their degradation is very slow.

● SMPs can be removed from effluents using a variety of different technologies, but the most effective process is adsorption by granular activated carbon.

● Discharging the SMPs could have deteriorating consequences on environment which needs developing the treatment processes undertaken for their removal. The solution lies on a collaboration between biologists and engineers. Biologists should generate more information on their production mechanisms, nature and properties, while the process engineers should be able to propose some solutions for their treatment in industrial plant scale.

## Competing interests

The authors declare that they have no competing interests.

## Authors’ contributions

This work was a part of HA’s master thesis focusing on “Major parameters affecting membrane fouling in MBRs” performed under MRM and MHS supervision. All three authors tried to present their experiences on this subject in combination with a comprehensive literature review. All authors read and approved the final manuscript.
